# High-Resolution Time-Frequency Spectrum-Based Lung Function Test from a Smartphone Microphone

**DOI:** 10.3390/s16081305

**Published:** 2016-08-17

**Authors:** Tharoeun Thap, Heewon Chung, Changwon Jeong, Ki-Eun Hwang, Hak-Ryul Kim, Kwon-Ha Yoon, Jinseok Lee

**Affiliations:** 1 Department of Biomedical Engineering, Wonkwang University School of Medicine, 460 Iksandeaero, Iksan, Jeonbuk 570-749, Korea; bami1314@wku.ac.kr (T.T.); heewon1001@wku.ac.kr (H.C.); 2 Imaging Science based Lung and Bone Disease Research Center, Wonkwang University, 460 Iksandeaero, Iksan, Jeonbuk 570-749, Korea; mediblue@wku.ac.kr; 3 Department of Internal Medicine, Wonkwang University School of Medicine, 460 Iksandeaero, Iksan, Jeonbuk 570-749, Korea; eyesmile@wku.ac.kr (K.-E.H.); kshryj@wku.ac.kr (H.-R.K.); 4 Department of Radiology, Wonkwang University School of Medicine, 460 Iksandeaero, Iksan, Jeonbuk 570-749, Korea

**Keywords:** smartphone microphone, pulmonary function test, FEV_1_/FVC, COPD, high-resolution time-frequency

## Abstract

In this paper, a smartphone-based lung function test, developed to estimate lung function parameters using a high-resolution time-frequency spectrum from a smartphone built-in microphone is presented. A method of estimation of the forced expiratory volume in 1 s divided by forced vital capacity (FEV_1_/FVC) based on the variable frequency complex demodulation method (VFCDM) is first proposed. We evaluated our proposed method on 26 subjects, including 13 healthy subjects and 13 chronic obstructive pulmonary disease (COPD) patients, by comparing with the parameters clinically obtained from pulmonary function tests (PFTs). For the healthy subjects, we found that an absolute error (AE) and a root mean squared error (RMSE) of the FEV_1_/FVC ratio were 4.49% ± 3.38% and 5.54%, respectively. For the COPD patients, we found that AE and RMSE from COPD patients were 10.30% ± 10.59% and 14.48%, respectively. For both groups, we compared the results using the continuous wavelet transform (CWT) and short-time Fourier transform (STFT), and found that VFCDM was superior to CWT and STFT. Further, to estimate other parameters, including forced vital capacity (FVC), forced expiratory volume in 1 s (FEV_1_), and peak expiratory flow (PEF), regression analysis was conducted to establish a linear transformation. However, the parameters FVC, FEV1, and PEF had correlation factor *r* values of 0.323, 0.275, and −0.257, respectively, while FEV_1_/FVC had an *r* value of 0.814. The results obtained suggest that only the FEV1/FVC ratio can be accurately estimated from a smartphone built-in microphone. The other parameters, including FVC, FEV1, and PEF, were subjective and dependent on the subject’s familiarization with the test and performance of forced exhalation toward the microphone.

## 1. Introduction

Lung diseases are some of the most common medical conditions [1]. Many people suffer from lung diseases stemming from smoking, infections, and genetics. The lungs are the primary organs expanding and relaxing thousands of times each day to bring in oxygen and push out carbon dioxide. As one inhales, the breath travels through the mouth or nose to the windpipe and lungs, and further moves through smaller tubes to deliver oxygen throughout the organ. A number of lung diseases adversely affect lung function by affecting the airways. These include asthma, chronic obstructive pulmonary disease (COPD), chronic bronchitis, emphysema, acute bronchitis, and cystic fibrosis [2].

**Figure 1:**
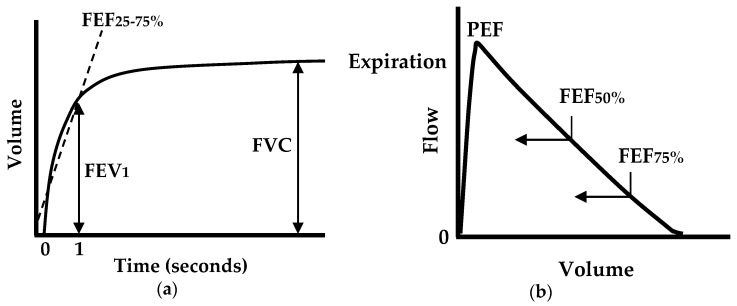
Examples of forced expiratory flow measure, (**a**) volume-time plot; and (**b**) volume–flow plot [7].

For lung function evaluation, pulmonary function tests (PFTs) have found widespread clinical use. PFTs can help diagnose asthma, allergies, chronic bronchitis, respiratory infections, lung fibrosis, bronchiectasis, and COPD by evaluating how well the lungs take in and release air. For basic PFTs, a spirometer is the main piece of equipment, and an important clinical diagnostic device that should be used by all primary care and most specialist physicians [3]. Indeed, it was reported that 66% of primary care offices owned and used their own spirometer at the primary contact point. This low amount should reflect, in part, the need for alternatives to the spirometer [4,5]. A standard spirometer measures forced expiratory flow and displays a volume-time curve and a flow–volume loop (or curve), as shown in Figure 1. From the measurements, the following parameters are quantized: the forced vital capacity (FVC); the forced expiratory volume in one second (FEV_1_); the FEV_1_/FVC ratio; the peak expiratory flow (PEF); the forced expiratory flow at 25%, 50%, or 75% of FVC (FEF_25_, FEF_50_, and FEF_75_); and the forced expiratory flow between 25% and 75% (FEF_25%–75%_) [6,7,8]. All of these parameters are clinically important in assessing lung function.

Among these parameters, the most common clinical measures are FVC, FEV_1_, FEV_1_/FVC, and PEF, as they are used to quantify the degree of airflow limitation in chronic lung diseases, such as asthma, COPD, and cystic fibrosis. The FEV_1_/FVC ratio has been widely accepted, especially for assessing COPD, which obstructs the main airway. In 1983, the European Community for Coal and Steel (ECCS) initially defined airway obstruction with an FEV_1_/FVC ratio below the lower fifth percentile of a large healthy reference group, which is the statistically-defined lower limit of normal (LLN) [9]. The same definition was followed by the American Thoracic Society (ATS) in 1991 [10], the European Respiratory Society (ERS) in 1993 [11], and the National Lung Health Education Program (NLHEP) in 2000 [12]. Depending on the organization and year, the definition has occasionally been modified by using a fixed value instead of the LLN. In 1987, ATS defined airway obstruction with an FEV_1_/FVC ratio lower than the fixed value of 0.75 [13], followed by the British Thoracic Society (BTS) with 0.70 in 1997 [14], the National Institute for Health and Clinical Excellence (NICE) with 0.75 in 2004 [15], the ATS and ERS with 0.70 in 2004 [16], and the Global Initiative for Chronic Obstructive Lung Disease (GOLD) with 0.75 in 2007 [15]. In this way, the FEV_1_/FVC ratio has been a widely accepted standard for assessing COPD, as summarized in Table 1 [15].

Home spirometry may provide the convenient way for patients who live great distances from their clinics and research facilities. It also allows patients to monitor more frequently for detecting changes in lung function, which results in in earlier treatment of exacerbations, more rapid recovery, reduced healthcare cost, and improved outcomes [17,18]. Even though there are portable spirometers available, they are expensive, which limits the popularity to many people. Since the smartphone is popular and capable for users to measure forced exhalation with its built-in microphone, it can be a cheap home spirometer available to smartphone users. In addition, since smartphone technology has the capacity for users to communicate with physicians (e.g., using cloud technology), smartphone-based home spirometry has the potential in providing early diagnosis of lung disease for those who may have breathing problems on a day-to-day basis [19,20,21,22,23]. Recently, a smartphone-based approach that measures lung function using the built-in microphone has been introduced [24]. In their pilot study, the researchers demonstrated the extraction of the FVC, FEV_1_, PEF, and FEV_1_/FVC parameters. However, the approach used the spectrogram of the signal based on the short-time Fourier transform (STFT) with 30 ms frames. The STFT has a limitation in its time-frequency resolution capability, which is due to the uncertainty principle. The Heisenberg-Gabor inequality states that the time–bandwidth product of a signal is the lower bound by some constant. This means that a signal cannot be simultaneously narrow in time and frequency domains. These limitations in resolution were one of the reasons for the invention of wavelet theory. Recently, the variable frequency complex demodulation method (VFCDM) has shown a higher resolution than any other time-frequency spectrum methods, such as the smoothed pseudo Wigner-Ville (SPWV), short-time Fourier transform (STFT), and the wavelet transform (WT) methods [25,26,27,28]. 

In this paper, VFCDM-based lung function parameter estimation is presented by using an audio signal recorded from a smartphone built-in microphone. The estimation method of the FEV_1_/FVC ratio is first presented, and the accuracy of the VFCDM is then compared with that of CWT and STFT. The main contribution is to introduce the smartphone built-in microphone-based FEV_1_/FVC ratio estimation by using the high-resolution time-frequency spectrum, VFCDM, which accurately detects the main time-varying resonance frequency. To evaluate our proposed method, we compared the estimated FEV_1_/FVC ratio with the parameters clinically obtained from PFTs. In addition, we evaluated the other parameter estimation of FVC, FEV_1_, and PEF.

## 2. Materials and Methods 

### 2.1. Subjects

Twenty-six subjects took part in this study, including 13 healthy subjects and 13 COPD patients. The healthy subjects (10 males, three females), with ages ranging from 24–47 years (30.85 ± 7.74; mean ± STD), had an average weight of 68.15 ± 16.01 kg and an average height of 170.23 ± 9.18 cm. The COPD patients (10 males, three females), with ages ranging from 51–89 years (71.92 ± 10.49), had an average weight of 61.00 ± 10.54 kg and an average height of 163.00 ± 6.30 cm. They were all severe COPD. Table 2 lists the subject details including their average lung function parameters. The study protocol and data analysis were approved by the Institutional Review Board (IRB) at Wonkwang University Hospital (WKUH), and all subjects consented to participate in the experiment. Since the forced expiration could aggravate certain medical conditions, individuals with the following medical conditions were excluded: hemoptysis of unknown origin; pneumothorax; angina; recent myocardial infarction; thoracic, abdominal, and cerebral aneurysms; cataracts or recent eye surgery; recent thoracic or abdominal surgery; nausea; vomiting; acute illness; and recent viral infection.

### 2.2. Data Collection and Preprocessing

The participants were instructed to follow the rules before the spirometry test [29]:

- Do not smoke during the hour before the test;
- Do not drink alcohol during the four hours before the test;
- Do not eat a large meal during the two hours before the test;
- Please wear loose clothing;
- Do not perform vigorous exercise within 30 min of the test; and
- If on puffer (inhaler) medication, you may be asked to refrain from taking it for a few hours before the test.

**Figure 2:**
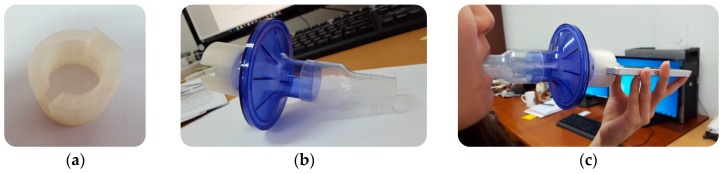
Audio data acquisition setup: (**a**) 3D-printing mouthpiece-to-iPhone adapter; (**b**) mouthpiece equipped with adapter; and (**c**) complete set of experimental and data acquisition equipment.

The audio data was measured and collected using an iPhone 5S (manufactured by Apple Inc., Cupertino, CA, USA). The subjects’ forced exhalation sounds were recorded using the built-in microphone and converted into digital audio signals at a sampling rate of 44,100 Hz. The subjects were required to breathe in their full lung capacity and then forcefully exhale as much air from the lungs as possible through the mouthpiece (normally taking over 6 s). The mouthpiece was attached to the iPhone microphone using a custom-made 3D-printed adapter (see Figure 2a,b). To achieve the spirometry measurement, much effort has been carried out on the vortex whistle [30,31,32,33]. We modified the vortex whistle available for the smartphone built-in microphone. The mouthpiece used in this experiment was VBMax PFT Filter 33 mm from A-M Systems (Sequim, WA, USA). The adapter was printed with polylactic acid (PLA) material. It is an end-opened device which allows the air to pass through to the microphone and exit freely at the end. Rubber material was inserted between the mouthpiece and an adapter to prevent air leakage. The mouthpiece plays an important role because it essentially helps to maintain lip posture, fixes the distance between the lips and the microphone, and reduces surrounding background noise by guiding the airflow directly towards the microphone. Furthermore, it attaches and detaches easily to an iPhone 5S for an older person, and enables the test to be performed in the same manner as that performed with clinical equipment at a hospital. The clinical test was performed at Wonkwang University Hospital and the smartphone-based test was performed in an open-space environment. Participants performed the clinical tests followed by smartphone-based test under two trained pulmonologists. Performing clinical test at first allows participants to familiarize with the smartphone-based test. Participants performed three trial tests with the smartphone, as shown in Figure 2c. Each test was completely effort-dependent, and each participant was instructed by a trained specialist using gestures. The original audio signals with a sampling rate of 44,100 Hz were down-sampled to 2450 Hz, and a seventh-order elliptic low-pass filter with a cutoff frequency of 800 Hz was applied to remove unwanted signal components. These parameters were found based on the prior-evaluation tests under a very quiet room without any noise interference. In addition, to reduce the computational complexity, we down-sampled the signal to 2450 Hz, which still preserved the recorded data. Among the three trials from the smartphone-based test, the parameter set was selected based on the Morris/Polgar standards [34,35,36,37] under the two trained pulmonologists. The participants also performed three trial tests with clinical equipment as a reference, with some times between tests to allow recovery to a rested condition. The pulmonary function test (PFT) data was captured using Vmax software version IVS-0101-21-2B (manufactured by CareFusion Corporation, San Diego, CA, USA), which automatically provided the PFT parameters. Each participant performing PFT was seated in the cabin of a Vmax Autobox (Body Plethysmography System, V62J) manufactured by CareFusion, and performed the test. 

### 2.3. Resonance Frequency Estimation Using High Resolution Time-Frequency Spectrum

Using the audio signal, we analyzed the dominant frequency of the forced exhalation sound by using the variable frequency complex demodulation (VFCDM) method [26]. The VFCDM method has shown higher resolution than any other time-frequency spectrum methods, such as smoothed pseudo Wigner-Ville (SPWV), short-time Fourier transform (STFT) and wavelet transform (WT) methods [25,26,27,28]. Considering a sinusoidal signal *x*(*t*) to be a narrow band oscillation with an instantaneous amplitude *A*(*t*), a center frequency $f_{0}$, a phase *ϕ*(*t*), and a direct current component *dc*(*t*):

(1) $$x\left(t\right)=dc\left(t\right)+A\left(t\right)\cos\left(2\pi f_{0}t+\varphi \left(t\right)\right)$$

For a given center frequency, information on the instantaneous amplitude *A*(*t*) and phase *ϕ*(*t*) are extracted by multiplying Equation (1) by $e^{-j2\pi f_{0}t}$, providing the following formula:

(2) $$z\left(t\right)=x\left(t\right)e^{-j2\pi f_{0}t}=dc\left(t\right)e^{-j2\pi f_{0}t}+\frac{A\left(t\right)}{2}e^{j\varphi \left(t\right)}+\frac{A\left(t\right)}{2}e^{-j\left(4\pi f_{0}t+\varphi \left(t\right)\right)}$$

The shift by $e^{-j2\pi f_{0}t}$ results in moving the center frequency, $f_{0}$, to zero frequency in the spectrum of *z*(*t*). If *z*(*t*) in Equation (2) is subjected to an ideal low-pass filter (LPF) with a cutoff frequency $f_{c}$ *<* $f_{0}$, the filtered signal $z_{lp}\left(t\right)$ contains only the component of interest, and the following formulae are obtained:

(3) $$z_{lp}\left(t\right)=\frac{A\left(t\right)}{2}e^{j\varphi \left(t\right)}$$

(4) $$A\left(t\right)=2\left|z_{lp}\left(t\right)\right|$$

(5) $$\varphi \left(t\right)=\;dc\left(t\right)+A\left(t\right)\cos\left(\int_{0}^{t}2\pi f\left(\tau \right)d\tau +\varphi \left(t\right)\right)$$

The acoustic signal *x*(*t*) contains the modulating frequency, which changes according to time. Therefore, *x*(*t*) can be formulated as:

(6) $$x\left(t\right)=dc\left(t\right)+A\left(t\right)\cos\left(\int_{0}^{t}2\pi f\left(\tau \right)d\tau +\varphi \left(t\right)\right)$$

Similarly, multiplying Equation (6) by $e^{-j\int_{0}^{t}2\pi f\left(\tau \right)d\tau }$ provides the instantaneous amplitude, *A*(*t*), and instantaneous phase, *ϕ*(*t*):

(7) $$z\left(t\right)=x\left(t\right)e^{-j\int_{0}^{t}2\pi f\left(\tau \right)d\tau }=dc\left(t\right)e^{-j\int_{0}^{t}2\pi f\left(\tau \right)d\tau }+\frac{A\left(t\right)}{2}e^{j\varphi \left(t\right)}+\frac{A\left(t\right)}{2}e^{-j\left(\int_{0}^{t}4\pi f\left(\tau \right)d\tau +\varphi \left(t\right)\right)}$$

From Equation (7), when *z*(*t*) is filtered with an ideal LPF with a cutoff frequency $f_{c}$ < $f_{0}$, the filtered signal $z_{lp}\left(t\right)$ contains the same instantaneous amplitude *A*(*t*) and phase *ϕ*(*t*) as in Equations (4) and (5). Details regarding the VFCDM algorithm are described in [26].

**Figure 3:**
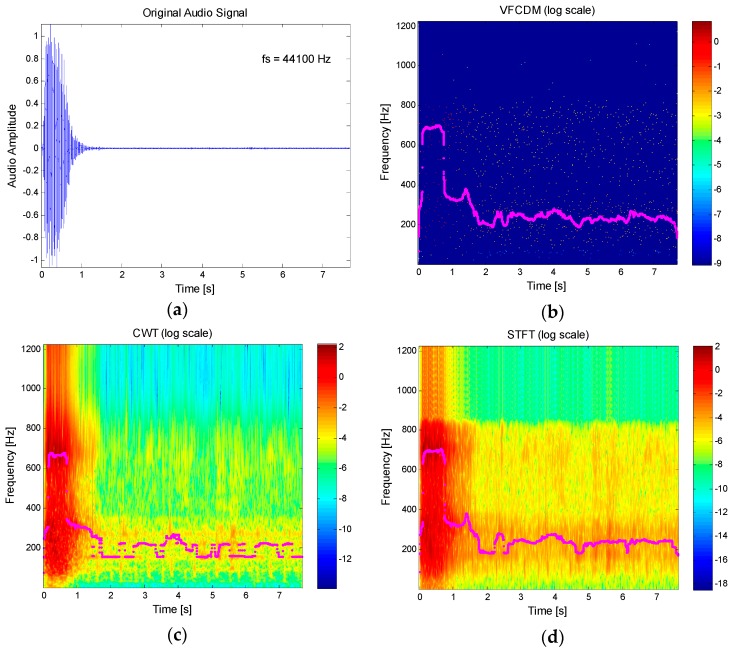

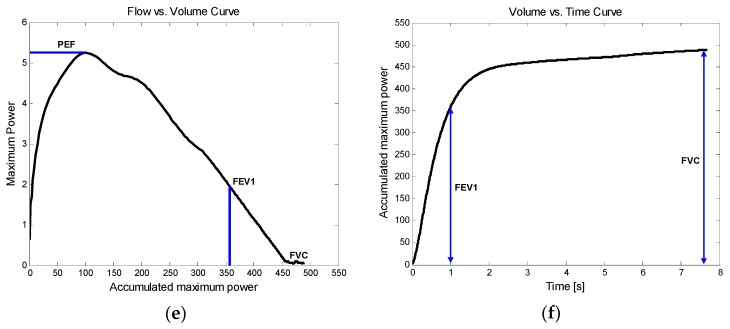
Main procedure to obtain flow-volume curve and volume-time curve from healthy subjects and comparison of VFCDM, CWT, and STFT: (**a**) original audio signal; (**b**) resultant time-frequency spectrum from VFCDM; (**c**) resultant time-frequency spectrum from CWT; (**d**) resultant time-frequency spectrum from STFT; (**e**) maximum power vs. accumulated maximum power from VFCDM; and (**f**) accumulated maximum power vs. time from VFCDM.

Figure 3 shows an example of the main procedure to obtain the volume-flow curve and time-volume curve from the audio signal, as well as the comparison among the resultant time-frequency spectra of VFCDM, CWT, and STFT. Figure 3a shows the original audio signal with a sampling rate of 44,100 Hz from the built-in microphone. The original audio signal was then down-sampled to 2450 Hz, and the seventh-order elliptic LPF with a cutoff frequency of 800 Hz was applied. Figure 3b**–**d show the resultant time-frequency spectra using VFCDM, CWT, and STFT, respectively. The filter parameters of the VFCDM were with the bandwidth *F_w_* = 0.03 Hz (normalized frequency) and the length of the filter *N_ω_* = 64. For CWT, the Morlet wavelet scalogram was chosen with the lowest and highest normalized frequencies set to 0.01 Hz and 0.5 Hz. Morlet wavelet transform allows multi-resolution analysis in the time-frequency domain of a non-stationary, transient signal. For STFT, the data was buffered into 30 ms frames and shifted by every sample. Each frame is then windowed using a Hamming window and then computed the magnitude squared of the FFT to produce a spectrogram. In Figure 3b,d the frequencies providing the maximum power for each sample, with an interval of 1/2450 s, are also plotted. These show that VFCDM provides a higher resolution in both time and frequency than CWT and STFT. With the time-frequency spectrum from VFCDM, we plotted maximum power versus accumulated maximum power, as shown in Figure 3e, which shows a similar pattern to the flow-volume curve depicted in Figure 1b. In addition, we plotted the accumulated maximum power versus the sample (time), as shown in Figure 3f, which shows a similar pattern to the volume-time curve depicted in Figure 1a.

### 2.4. Instantaneous Power and Performance Evaluation

VFCDM, CWT, and STFT were applied for the estimation of the FEV_1_/FVC ratio for healthy subjects (*N* = 13) and COPD patients (*N* = 13). To investigate the effect of instantaneous power, the summation of power values equal to or greater than a varying percentage *TH_P_* of the maximum power at each sample was considered. *TH_P_* was altered to 50%, 70%, 80%, 90%, and 100%, and the estimated FEV_1_/FVC ratio was evaluated. Using the power summation for each sample, power summation vs. accumulated power summation was plotted representing the flow–volume curve, and time vs. accumulated power summation was plotted representing the volume-time curve. 

To further investigate the VFCDM based estimation of the other lung function parameters including FVC, FEV_1_, and PEF, the values of total power, total power in 1 s, and global maximum power were extracted, each of which corresponds to FVC, FEV_1_, and PEF, respectively. The extracted parameters are denoted by $S_{FVC}$, $S_{FEV1}$, and $S_{PEF}$. It was assumed that $S_{FVC}$, $S_{FEV1}$, and $S_{PEF}$ were related by the following linear transformation:

(8) $$T_{FVC}\approx S_{FVC}=K_{FVC}\cdot E_{FVC}+C_{FVC}$$

(9) $$T_{FEV1}\approx S_{FEV1}=K_{FEV1}\cdot E_{FEV1}+C_{FEV1}$$

(10) $$T_{PEF}\approx S_{PEF}=K_{PEF}\cdot E_{PEF}+C_{PEF}$$

where $T_{FVC}$, $T_{FEV1}$, and $T_{PEF}$ are the reference values, and $S_{FVC}$, $S_{FEV1}$, and $S_{PEF}$ are the estimated values of FVC, FEV_1_, and PEF, respectively. All the reference values were obtained from the clinical results. $E_{FVC}$ is the summation of maximum power (*TH_P_* = 100%) for the entire recorded samples, $E_{FEV1}$ is the summation of maximum power for the first one second, and $E_{PEF}$ is the global maximum power in the entire recorded samples; $K_{FVC}$, $K_{FEV1}$, and $K_{PEF}$ are the scaling constants; and $C_{FVC}$, $C_{FEV1}$, and $C_{PEF}$ are offset constants. For each equation, we have a dependent variable corresponding to a reference value and an independent variable corresponding to a predictor. Then, the relationship with the constant values between a dependent variable ($T_{FVC}$, $T_{FEV1}$, or $T_{PEF}$) and an independent variable ($E_{FVC}$, $E_{FEV1},$ or $E_{PEF}$) can be estimated by linear least squares regression. Statistical analyses were performed using IBM SPSS Statistics for Windows, version 22.0 (manufactured by IBM Corp., Armonk, NY, USA, 2012). Pearson’s correlation coefficients were used for performing linear regression analysis. For comparison with the correlation coefficient from the FEV_1_/FVC ratio, we similarly formulated the linear transformation as:

(11) $$T_{FEV1/FVC}\approx S_{FEV1/FVC}=K_{FEV1/FVC}\cdot E_{FEV1/FVC}+C_{FEV1/FVC}$$

where $E_{FEV1/FVC}=E_{FEV1}/E_{FVC}$.

For the evaluation, we used absolute error (AE) and root mean squared error (RMSE) defined by:

(12) $$\text{Absolue error }\left(\text{AE}\right)=\;\left|E_{\text{FEV}1/\text{FVC}}-\;T_{\text{FEV}1/\text{FVC}}\right|$$

(13) $$\text{Root Mean Squared Error }\left(\text{RMSE}\right)=\;\sqrt{\sum \frac{{\left(E_{\text{FEV}1/\text{FVC}}-\;T_{\text{FEV}1/\text{FVC}}\right)}^{2}}{N}}$$

where *N* is the number of subjects; and $E_{\text{FEV}1/\text{FVC}}$ and $T_{\text{FEV}1/\text{FVC}}$ are the estimated FEV_1_/FVC ratio and its reference value, respectively. 

## 3. Results

### 3.1. Estimation of FEV_1_/FVC Ratio

**Figure 4:**
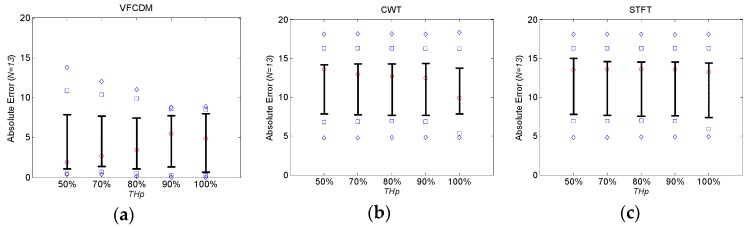
Absolute error distribution of FEV_1_/FVC from healthy subjects according to *TH_P_* based on (**a**) VFCDM; (**b**) CWT; and (**c**) STFT.

Figure 4a–c show the absolute error distributions of the estimated FEV_1_/FVC ratios from healthy subjects according to *TH_P_* based on VFCDM, CWT and STFT, respectively. The central red circle mark is the median, the lower and upper whiskers are the 25th and 75th percentiles, the blue square marks are the 10th and 90th percentiles, and the blue diamond marks are the 5th and 95th percentiles. A significant difference between VFCDM and CWT at *p* < 0.01 was found based on the *t*-test. Similarly, a significant difference between VFCDM and STFT was found. In addition, it was found that the absolute error decreased as *TH_P_* increased up to 100% for all VFCDM, CWT, and STFT. Thus, VFCDM with *TH_P_* = 100% provided the best accuracy for the FEV_1_/FVC ratio estimation. Table 3 summarizes the absolute error (AE) and RMSE values from healthy subjects according to *TH_P_* for VFCDM, CWT, and STFT, respectively. It shows that RMSE from VFCDM with *TH_P_* = 100% is 2.07 and 2.19 times lower than the CWT and STFT with *TH_P_* = 100%, respectively. For mean AE, VFCDM with *TH_P_* = 100% provided 2.39 and 2.57 times lower than CWT and STFT with *TH_P_* = 100%, respectively.

**Figure 5:**
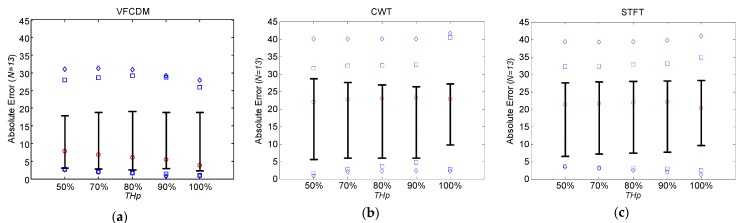
Absolute error distribution of FEV_1_/FVC from COPD patients according to *TH_P_* based on (**a**) VFCDM; (**b**) CWT; and (**c**) STFT.

Figure 5a–c show the absolute error distributions of the estimated FEV_1_/FVC ratios from COPD patients according to *TH_P_* based on VFCDM, CWT and STFT, respectively. A significant difference between VFCDM and CWT at *p* < 0.01 was found based on the *t*-test. Similarly, a significant difference between VFCDM and STFT was found. Table 4 summarizes the absolute error (AE) and RMSE values from COPD subjects according to *TH_P_* for VFCDM, CWT, and STFT, respectively. It shows that RMSE from VFCDM with *TH_P_* = 100% is 1.67 and 1.58 times lower than the CWT and STFT with *TH_P_* = 100%, respectively. For mean AE, VFCDM with *TH_P_* = 100% provided 2.03 and 1.91 times lower than CWT and STFT with *TH_P_* = 100%, respectively.

Table 5 summarizes the whole estimated FEV_1_/FVC ratios from each subject at *TH_P_* = 100% for VFCDM, CWT, and STFT. For healthy subjects and COPD patients, the VFCDM provided higher accuracy. In addition, for healthy subjects, VFCDM showed reasonably accurate estimation of FEV_1_/FVC. However, for COPD patients, the estimation results were not satisfied with the clinical needs. Nevertheless, all of the COPD results with VFCDM were less than 0.70, which met the criteria for assessing COPD summarized in Table 1.

**Figure 6:**
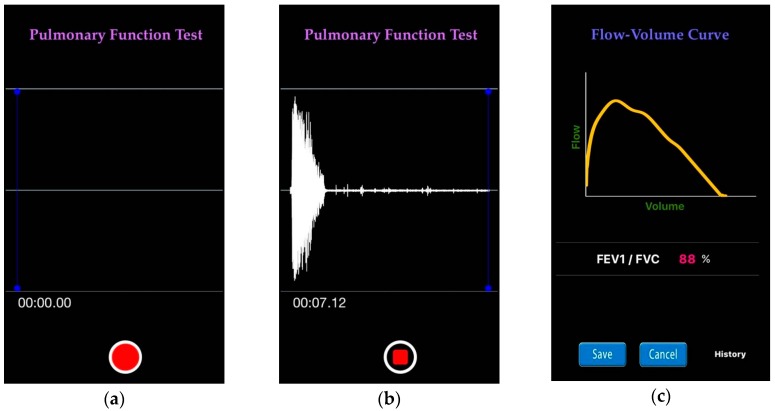
Snapshots of iPhone application for FEV_1_/FVC ratio estimation, developed using the Objective-C programming language: (**a**) before recording; (**b**) forced exhalation being recorded; and (**c**) flow-volume curve and estimated FEV_1_/FVC ratio displayed.

Figure 6a–c show screen snapshots of the iPhone 5S application for FEV_1_/FVC ratio estimation, developed using the Objective-C programming language. Once recording is completed, it takes five seconds for the app to visualize the FEV_1_/FVC ratio estimation result in real-time. Figure 6a shows the screen at the commencement of recording the forced exhalation through the built-in microphone. Figure 6b shows the forced exhalation in the process of being recorded. Recording can be stopped by clicking the circle button at the bottom of the screen. After the recording is complete, the flow-volume curve and the estimated FEV_1_/FVC ratio are displayed, as shown in Figure 6c.

### 3.2. Regression and Estimation of FVC, FEV_1_, and PEF

**Figure 7:**
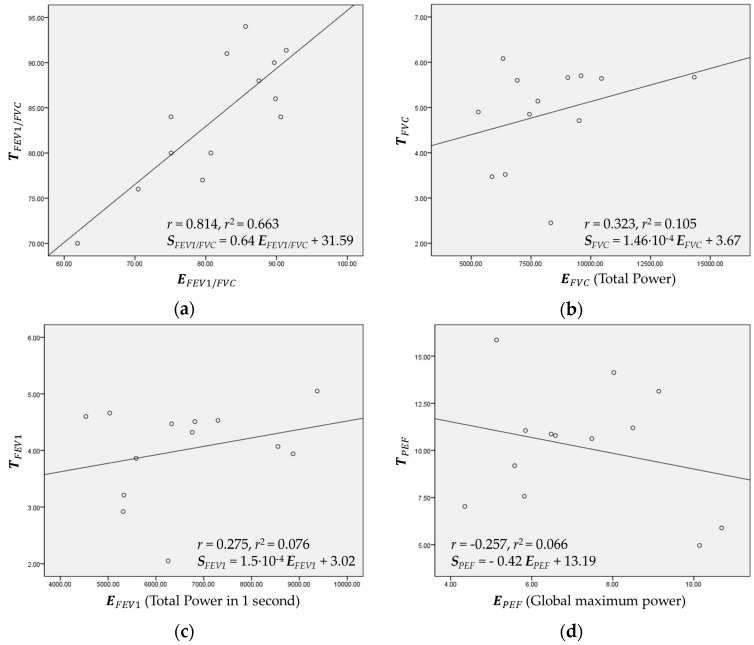
Regression plots of: (**a**) $E_{\text{FEV}1/\text{FVC}}$ vs. $T_{\text{FEV}1/\text{FVC}}$, (**b**) $E_{\text{FVC}}$ vs. $T_{\text{FVC}}$, (**c**) $E_{\text{FEV}1}$ vs. $T_{\text{FEV}1}$; and (**d**) $E_{\text{PEF}}$ vs. $T_{\text{PEF}}$.

Figure 7a–d show the regression plots based on VFCDM for FEV_1_/FVC, FVC, FEV_1_, and PEF, respectively, from healthy subjects. As expected, in Figure 7a, $E_{FEV1/FVC}$, and $T_{FEV1/FVC}$ were linearly correlated with *r* = 0.814. The constants $K_{FEV1/FVC}$ and $C_{FEV1/FVC}$ were found to be 0.64 and 31.59, respectively. On the other hand, as shown in Figure 7b–d, FVC, FEV_1_, and PEF did not show strong correlation, respectively. 

## 4. Discussion

We presented a smartphone-based lung function test using a high-resolution time-frequency spectrum from a smartphone built-in microphone. Even though the time-frequency spectrum is not a sole component for the result accuracy, it is the key algorithm to increase the accuracy. In [24], the audio from a phone was buffered into 30 ms frames and each frame was windowed to quantify the magnitude spectrogram of the signal. On the other hand, we down-sampled to 2450 Hz and visualized the time-frequency spectrum with the time interval of 1/2450 s, which is equivalent to 0.41 ms, approximately. Hence, our method has approximately 73 times higher time resolution than the previous work. More specifically, to estimate the FEV_1_/FVC ratio, VFCDM provided lower absolute errors than CWT and STFT by 6.52 and 6.67, respectively. In addition, VFCDM provided lower RMSEs than CWT and STFT by 5.94 and 6.62, respectively. The results suggest that the VFCDM approach provided higher accuracy of the FEV_1_/FVC ratio than CWT and STFT due to high resolution of the time-frequency spectrum. However, we also found that only the FEV_1_/FVC ratio can be accurately estimated using the smartphone built-in microphone since the ratio as a relative value can be obtained directly without the estimates of FVC and FEV_1_. These two factors, as well as PEF, are subjective and dependent on the subject’s familiarization with the test and performance of the forced exhalation. More specifically, each individual may exhale with slightly different angles toward a built-in microphone even with the add-on mouthpiece. In addition, each individual may bite the mouthpiece deeply or shallowly. Each individual may also move or shake the smartphone during the forced exhalation test. Those factors affect the time-frequency spectrum, and may result in inaccurate estimation of FVC, FEV_1_, and PEF. Healthy subjects #2, #9, and #11 embodied such cases. Regarding FEV_1_/FVC, if the three subjects #2, #9, and #11 were excluded, the estimation results from the ten other healthy subjects provided very low absolute errors and RMSEs of 3.573 and 4.760, respectively. Regarding FVC, FEV_1_, and PEF, the correlation *r* value could increase to 0.763, 0.928, and 0.823, respectively. Thus, the limited and cautious force of exhalation toward the built-in microphone is necessary to increase the estimation accuracy. Furthermore, the future research needs to be toward operation without the add-on adapter. Furthermore, the additional condition needs to be considered: each individual should keep the distance constant between a lip and a microphone. Then, a user may have a trouble keeping the fixed distance between the lip and microphone before the test every time.

We also found that AE and RMSE of FEV_1_/FVC ratios from COPD patients were relatively high with 10.30% ± 10.59% and 14.48%, respectively, even with VFCDM. These high errors were mainly from the artifact sound caused by the narrow windpipe, especially for COPD patients. Most COPD patients have a narrow windpipe, which causes artifact sound even in routine life. In the case of forced exhalation, the artifact sound is more dominating and the real forced exhalation sound is severely affected by the artifacts. Then, the time-varying main frequency cannot be accurately detected, and eventually the instantaneous frequency with maximum power at each sample cannot appropriately represent the volume of the PFT test. More specifically, in the portion of the sound without the artifacts, our proposed method with VFCDM provides higher accuracy than CWT and STFT. In the portion of the sound with the artifacts, there is no difference among VFCDM, CWT, and STFT. This reflects that the results from healthy subjects have higher accuracy than those from COPD patients. In addition, this reflects that the results with VFCDM have higher accuracy than CWT and STFT for both healthy subjects and COPD patients. Thus, the dispersion trend according to *TH_p_* is observed only in healthy subjects as shown in Figure 4 and Figure 5. More research regarding artifact sound detection and filtering should be directed to be clinically available in the future. 

In our study, among the healthy subjects, seven subjects were aged between 20 and 29 (group A), and the other six subjects were aged between 30 and 39 (group B). For groups A and B, RMSE values were 6.13 and 4.76, and AE values were 4.96 ± 3.89 and 3.93 ± 2.93. However, due to a small number of subjects, the effect of age is not clear from the results. Similarly, for male (*N* = 10) and female (*N* = 3) groups from healthy subjects, RMSE values were 4.61 and 7.90, and AE values were 3.48 ± 3.18 and 7.84 ± 1.17. From COPD patients, RMSE values were 15.17 and 11.92, and AE values were 10.65 ± 11.38 and 9.14 ± 99.36, respectively. Similarly with the gender effect, due to a small number of subjects and patients, the effect of gender is not clear from the results. Thus, further studies need to rigorously validate the FEV_1_/FVC ratio and revise the regression model for FVC, FEV_1_, and PEF by considering a larger number of subjects with age and gender matching.

## 5. Conclusions

In this paper, we developed to estimate lung function parameters using a high-resolution time-frequency spectrum from a smartphone built-in microphone. For both healthy and COPD groups, we evaluated the estimation performance of FEV_1_/FVC, and found that VFCDM was superior to CWT and STFT. Further, regression analysis was conducted to establish a linear transformation on FVC, FEV_1_ and PEF. However, correlation factor values were not high comparing to FEV_1_/FVC since they were subjective and dependent on the subject’s familiarization with the test and performance of forced exhalation toward the built-in microphone.

## Figures and Tables

**Table 1:** Criteria for assessing COPD according to various organizations [15].

Organization	Year	Criterion/Reference
ECCS	1983	FEV_1_/FVC < LLN [9]
ATS	1987	FEV_1_/FVC < 0.75 [13]
ATS	1991	FEV_1_/FVC < LLN [10]
ECCS/ERS	1993	FEV_1_/FVC < LLN [11]
BTS	1997	FEV_1_/FVC < 0.70 and FEV_1_ < 80% predicted [14]
NLHEP	2000	FEV_1_/FVC < LLN and FEV_1_ < LLN [12]
NICE	2004	FEV_1_/FVC < 0.75 and FEV_1_ < 80% predicted [15]
ATS/ERS	2004	FEV_1_/FVC < 0.70 post-bronchodilator [16]
GOLD	2007	FEV_1_/FVC < 0.75 post-bronchodilator [15]

**Table 2:** Subjects’ information.

Subjects	Healthy	Patient	Total
	(*n* = 13)	(*n* = 13)	(*n* = 26)
Gender (*n*)	Male 10	Male 10	Male 20
	Female 3	Female 3	Female 6
Age (*years*)	30.85 ± 7.74	71.92 ± 10.49	51.35 ± 22.85
Height (*cm*)	170.23 ± 9.18	163.00 ± 6.30	166.58 ± 8.51
Weight (*kg*)	68.15 ± 16.01	61.00 ± 10.54	64.58 ± 13.77
FVC (*Liters*)	4.89 ± 1.09	2.64 ± 0.82	3.77 ± 1.49
FEV_1_ (*Liters*)	4.02 ± 0.84	1.25 ± 0.52	2.63 ± 1.57
PEF (*Liter/s*)	10.62 ± 3.09	2.97 ± 1.06	6.80 ± 4.51
FEV_1_/FVC (*%*)	87.77 ± 6.61	48.31 ± 15.32	65.54 ± 21.03

Values are means ± standard deviations.

**Table 3:** Healthy subjects’ absolute errors (AE) and RMSEs of FEV_1_/FVC ratio according to *TH_P_* for VFCDM, CWT, and STFT methods.

*TH_P_*	*50%*		70%		*80%*		*90%*		*100%*	
	*AE*	*RMSE*	*AE*	*RMSE*	*AE*	*RMSE*	*AE*	*RMSE*	*AE*	*RMSE*
VFCDM (*N* = 13)	4.29 ± 4.44	6.05	4.44 ± 3.94	5.84	4.54 ± 3.72	5.78	4.65 ± 3.39	5.68	4.49 ± 3.38	5.54
CWT (*N* = 13)	11.76 ± 4.09	12.40	11.65 ± 4.10	12.30	11.60 ± 4.10	12.25	11.55 ± 4.10	12.20	10.77 ± 4.14	11.48
STFT (*N* = 13)	11.86 ± 4.18	12.52	11.75 ± 4.18	12.42	11.71 ± 4.19	12.38	11.67 ± 4.2	12.35	11.43 ± 4.31	12.16

**Table 4:** COPD patients’ absolute errors (AE) and RMSEs of FEV_1_/FVC ratio according to *TH_P_* for VFCDM, CWT, and STFT methods.

*TH_P_*	*50%*		70%		*80%*		*90%*		*100%*	
	*AE*	*RMSE*	*AE*	*RMSE*	*AE*	*RMSE*	*AE*	*RMSE*	*AE*	*RMSE*
VFCDM (*N* = 13)	10.01 ± 10.56	14.26	10.29 ± 10.57	14.46	10.25 ± 10.63	14.47	10.17 ± 10.23	14.15	10.30 ± 10.59	14.48
CWT (*N* = 13)	18.85 ± 13.14	22.69	18.87 ± 12.79	22.52	18.85 ± 12.59	22.39	18.88 ± 12.39	22.32	20.93 ± 12.55	24.15
STFT (*N* = 13)	19.43 ± 11.95	22.56	19.37 ± 11.86	22.48	19.33 ± 11.90	22.47	19.34 ± 11.98	22.51	19.63 ± 12.14	22.83

**Table 5:** Individual estimation values of FEV1/FVC at *TH_P_* = 100% using VFCDM, CWT, and STFT methods.

Healthy	#1	#2 ^1^	#3	#4	#5	#6	#7	#8	#9 ^1^	#10	#11	#12 ^1^	#13
Reference	88	84	80	77	76	91	94	90	91	80	70	84	86
VFCDM	87.50	90.59	75.11	79.54	70.45	91.06	85.63	89.67	82.97	80.71	61.88	75.08	89.86
CWT	98.35	99.62	87.99	95.81	85.92	99.04	98.67	98.12	96.57	89.87	83.53	98.26	99.63
STFT	98.85	99.74	86.21	95.51	89.24	99.20	98.70	97.44	98.24	94.05	84.83	98.34	99.67
**COPD**	**#1 ^1^**	**#2**	**#3**	**#4**	**#5**	**#6**	**#7**	**#8**	**#9**	**#10 ^1^**	**#11**	**#12**	**#13 ^1^**
Reference	37	59	26	41	51	67	61	69	29	56	61	60	26
VFCDM	17.61	30.56	17.90	42.43	57.06	66.02	66.22	69.66	25.31	54.97	42.30	28.51	18.97
CWT	27.16	55.96	52.27	81.10	77.59	89.92	89.94	93.26	70.91	58.03	78.10	40.64	35.72
STFT	29.15	40.88	36.31	83.44	79.11	89.51	89.91	93.79	62.07	58.87	75.86	39.68	24.94

^1^ Female subject.

## Abbreviations

PFT	Pulmonary function test
FVC	Forced vital capacity
FEV_1_	Forced expiratory volume in 1 s
FEV_1_/FVC	Forced expiratory volume in 1 s/forced vital capacity
PEF	peak expiratory flow
ATS	American Thoracic Society
BTS	British Thoracic Society
ECCS	European Community for Coal and Steel
ERS	European Respiratory Society
GOLD	Global Initiative for Chronic Obstructive Lung Disease
LLN	Lower limit of normal
NICE	National Institute for Health and Clinical Excellence
VFCDM	Variable frequency complex demodulation method
CWT	Continuous wavelet transform
RMSE	Root mean squared error
STFT	short time Fourier transform
SPWV	Smoothed pseudo Wigner-Ville
COPD	Chronic obstructive pulmonary disease
